# Epigenetic age acceleration, neonatal morbidities, and neurobehavioral profiles in infants born very preterm

**DOI:** 10.1080/15592294.2023.2280738

**Published:** 2023-11-20

**Authors:** Uriel Paniagua, Barry M. Lester, Carmen J. Marsit, Marie Camerota, Brian S. Carter, Jennifer F. Check, Jennifer Helderman, Julie A. Hofheimer, Elisabeth C. McGowan, Charles R. Neal, Steven L. Pastyrnak, Lynne M. Smith, Sheri A. DellaGrotta, Lynne M. Dansereau, T. Michael O’Shea, Todd M. Everson

**Affiliations:** a Department of Epidemiology, Emory University Rollins School of Public Health, Atlanta, GA, USA; b Department of Pediatrics, Brown Alpert Medical School and Women and Infants Hospital, Providence, RI, USA; c Brown Center for the Study of Children at Risk, Brown Alpert Medical School and Women and Infants Hospital, Providence, RI, USA; d Department of Psychiatry and Human Behavior, Brown Alpert Medical School, Providence, RI, USA; e Gangarosa Department of Environmental Health, Emory University Rollins School of Public Health, Atlanta, GA, USA; f Department of Pediatrics-Neonatology, Children’s Mercy Hospital, Kansas City, MO, USA; g Department of Pediatrics, Wake Forest School of Medicine, Winston-Salem, NC, USA; h Department of Pediatrics, University of North Carolina School of Medicine, Chapel Hill, NC, USA; i Department of Pediatrics, University of Hawaii John A. Burns School of Medicine, Honolulu, HI, USA; j Department of Pediatrics, Corewell Health, Helen DeVos Children’s Hospital, Grand Rapids, MI, USA; k Department of Pediatrics, Harbor-UCLA Medical Center, Torrance, CA, USA

**Keywords:** Neonatal ageing, epigenetic clock, preterm infants, neurobehavior, neonatal morbidity

## Abstract

Epigenetic age acceleration is a risk factor for chronic diseases of ageing and may reflect aspects of biological ageing. However, few studies have examined epigenetic ageing during the early neonatal period in preterm infants, who are at heightened risk of developmental problems. We examined relationships between neonatal age acceleration, neonatal morbidities, and neurobehavioral domains among very preterm (<30 weeks gestation) infants to characterize whether infants with early morbidities or different neurobehavioral characteristics had accelerated or decelerated epigenetic ageing. This study uses data from the Neonatal Neurobehavior and Outcomes in Very Preterm Infants (NOVI) study, restricted to infants with data on variables assessed (*n* = 519). We used generalized estimating equations to test for differences in age acceleration associated with severe neonatal medical morbidities and neurobehavioral characteristics. We found that infants with neonatal morbidities, in particular, bronchopulmonary dysplasia (BPD), had accelerated epigenetic age – and some evidence that infants with hypertonicity and asymmetric reflexes had increased and decreased age acceleration, respectively. Adjustment for gestational age attenuated some associations, suggesting that the relationships observed may be driven by the duration of gestation. Our most robust finding shows that very preterm infants with neonatal morbidities (BPD in particular) exhibit age acceleration, but most neonatal neurobehavioral characteristics and morbidities are not associated with early life age acceleration. Lower gestational age at birth may be an upstream factor driving these associations.

## Introduction

Preterm birth, defined as birth prior to 37 weeks of gestation, accounts for nearly 10% of births in the United States and is the leading cause of death among children under 5 years of age [1]. The final weeks of pregnancy are a critical period of development for several vital organs [2], and experiencing this critical window *ex-utero* has consequences. Preterm births can be sub-categorized as moderate to late for those born between 32 and 37 weeks gestation, very preterm for those before 32 weeks gestation, and extremely preterm for those born less than 28 weeks gestation [3]. Infants born very preterm are at the highest risk for medical complications including bronchopulmonary dysplasia (BPD), severe brain injury, severe retinopathy of prematurity (ROP), and serious neonatal infections during hospitalization, which may negatively impact short- and long-term neurobehavioral outcomes [4–6]. As several studies have shown, even small differences in gestational age (time between conception and birth) can have significant impacts on neonatal outcomes and long-term neurodevelopmental impairments. These sub categorizations of prematurity are essential to appropriately evaluate neonatal risk for acute and chronic morbidities [7–10], highlighting the strong interrelationships between development, age, and health at birth and during the neonatal period.

Chronological age since conception (postmenstrual age [PMA]) strongly correlates with organ growth and development. However, even among infants with identical PMA, some may be more developmentally mature than others. Biological age, as opposed to chronological age, may reveal more about developmental states, but this is an elusive metric. Epigenetic age is an alternative age metric that is estimated based on DNA methylation (DNAm) levels and has been suggested as a tool that may capture aspects of biological ageing [11], but this is an ongoing area of study requiring further research, particularly in early childhood. Epigenetic age may be useful in assessing the health of infants that were born preterm as it may be an indicator of developmental maturity.

Several epigenetic clocks have been developed across different age groups and human tissues [12] in order to better understand the processes of ageing and to develop biomarkers of biological ageing and associated diseases. Horvath developed the first pan-tissue epigenetic clock by combining publicly available datasets measured on the Illumina 27K or Illumina 450K platforms. The clock utilizes 353 CpGs and has been shown to provide accurate estimates of age across different tissues and cell types [11,13]. Although estimates of epigenetic age or biological age derived using this clock have been shown to highly correlate with chronological age in adolescent and adult populations, the clock yields estimates that are more variable and not as accurate when applied to paediatric populations, as DNAm changes in early life may not be reflective of those later in life [13,14].

The Paediatric-Buccal-Epigenetic (PedBE) clock was developed to address gaps in DNAm in children and utilizes buccal epithelial cell DNAm at 94 CpG sites [14]. The clock focuses on the estimation of chronological age of children ranging from birth to 20 years old and was found to have less variability compared to the Horvath clock when applied to paediatric samples. Though the PedBE clock may yield better estimates than prior epigenetic clocks when applied to paediatric populations, the clock was derived using samples from typically-developing individuals between the ages of 0 and 20 years, and therefore may not be as reliable when applied to infants born very preterm and using samples collected during the neonatal period [14].

To further address gaps in methylation clocks for infants born very preterm (at <30 weeks gestation), we previously developed four NEOage clocks that predict postmenstrual age (PMA) and postnatal age (PNA; age since birth) using DNA methylation from 303 to 522 CpGs. PMA is an age metric that adds the gestational age at birth to the chronological age after birth (both in weeks) and is the preferred term for describing age during the NICU stay, among infants that were born preterm. Our clocks are compatible with the Illumina EPIC and 450k arrays and were developed using buccal epithelial cells obtained from 542 infants enrolled in the Neonatal Neurobehavior and Outcomes in Very Preterm Infants (NOVI) study [15]. Predicted age utilizing the NEOage clocks was found to be highly correlated with infant PMA and PNA during the early neonatal period [15]. Epigenetic clocks such as these have been utilized to gain insights into biological ageing through age acceleration, defined as the difference between chronological and epigenetic age. In younger populations, differences in epigenetic age acceleration have been linked to prenatal exposures including maternal anxiety, diet, body mass index (BMI), tobacco smoking status, as well as childhood psychiatric problems [16–20]. Epigenetic ageing studies of preterm infants, particularly during the early neonatal period, are limited.

We aimed to fill this gap by examining relationships between age acceleration, neonatal morbidities, and neurobehavioral domains among infants that were born very preterm. In this study, the neonatal morbidities are experienced during the NICU stay, while the neurobehavioral assessments and buccal swabs are collected just prior to discharge – thus our study characterizes epigenetic age acceleration that is concurrent with or in response to these characteristics. We utilized the NEOage epigenetic clocks for PMA and PNA compatible with the Illumina EPIC array and compared findings with two other epigenetic clocks – PedBE [14] and Horvath Skin-Blood [21], both of which included children’s buccal epithelial DNA in their training sets. The NEOage clock for PNA is the only clock to focus on postnatal age predictions in preterm infants, and yields predicted ages that correlate highly with reported ages [15]. Most research examining epigenetic age acceleration in preterm infants has utilized clocks derived from samples obtained from older subjects, which are less reliable when applied to individuals outside of the age range of the population used in clock development. The NEOage clocks derived from the NOVI cohort are expected to yield more reliable estimates of age acceleration for these very preterm infants. This paper aims to improve our current understanding of age acceleration during the early neonatal period and its associations with neonatal morbidities and neurobehavioral profiles and domains.

## Methods

### Study population

The Neonatal Neurobehavior and Outcomes in Very Preterm Infants (NOVI) study was conducted at nine university affiliated Neonatal Intensive Care Units (NICUs) in Providence (RI), Grand Rapids (MI), Kansas City (MO), Honolulu (HI), Winston Salem (NC), and Torrance and Long Beach (CA) from April 2014 through June 2016. These NICUs were also Vermont Oxford Network (VON) participants. Eligibility was determined based on the following inclusion criteria: 1) birth at <30 weeks postmenstrual age; 2) parental ability to read and speak English or Spanish; and 3) residence within 3 hours of the NICU and follow-up clinic. Exclusion criteria included maternal age <18 years, maternal cognitive impairment, maternal death, infants with major congenital anomalies, including central nervous system, cardiovascular, gastrointestinal, genitourinary, chromosomal, and non-specific anomalies, and NICU death. Parents of eligible infants were invited to participate in the study when survival to discharge was determined to be likely by the attending neonatologist. Overall, 704 eligible infants were enrolled, and buccal cells were collected on 624 of these infants for epigenomic screening. DNAm levels were profiled from buccal epithelial cells that were collected from study participants at NICU discharge using Illumina EPIC arrays. After quality-control (samples were excluded if there was insufficient DNA or arrays yielded poor QC metrics), pre-processing, and normalization as described in Graw et al. [15], samples from 542 individuals were available for analysis.

### NICU Neonatal Morbidities

To assess the impact of serious neonatal morbidities on age acceleration, we used an adaptation of Bassler et al.’s [4] validated cumulative neonatal morbidity risk score. The score was calculated by adding the total number of neonatal medical morbidities including BPD, severe brain injury, severe retinopathy of prematurity (ROP), and necrotizing enterocolitis (NEC) and/or culture-confirmed sepsis experienced by infants in the NOVI cohort during their NICU stay. Neonatal morbidity risk scores ranged from 0 to 4, although 3 and 4 were grouped into a single category (3+) due to a limited number of infants experiencing all four morbidities [22]. Morbidities were also examined separately to determine their specific associations with age acceleration. BPD was also available as a 4-level factor for representing severity: no BPD, mild BPD (supplemental oxygen or high-flow nasal cannula), moderate BPD (supplemental oxygen via high-flow nasal cannula, nasal continuous positive airway pressure, or nasal positive airway pressure ventilation), and severe BPD (mechanical ventilation via endotracheal tube) at ≥36 weeks PMA [6,23]. Due to small numbers of severe BPD, we combined moderate and severe BPD, and analysed this as a 3-level factor.

### NICU Network Neurobehavioral Scale (NNNS)

The NICU Network Neurobehavioral Scale (NNNS) is a standardized tool commonly used in research examining at-risk infants such as those born preterm [24]. This tool assesses infant neurobehavioral organization, neurological reflexes, motor development, and signs of stress and withdrawal [4]. Responses from the infant assessments were converted to 12 domain summary scores including attention, handling, self-regulation, arousal, excitability, lethargy, hypertonicity, hypotonicity, non-optimal reflexes, asymmetric reflexes, quality of movement and stress abstinence. Specifics about the individual domains are detailed in Lester et al. [24]. Our prior work in the NOVI cohort used latent profile analysis to identify six mutually exclusive neurobehavioral profiles based on these summary scores [22]. Those infants in profile 6 had the most poorly regulated response patterns, with more intense arousal predictive of non-optimal developmental outcomes in childhood [25,26]. Infants in Profile 5 also demonstrated poorly regulated patterns indicative of subdued attention, tone, movement, and arousal. Detailed methods of the neurobehavioral profiles are discussed in Everson et al. [22] and McGowan et al. [27]. This paper will focus on comparing the two most atypical neurobehavioral profiles − 5 and 6 – to infants in profiles 1–4 which exhibited more typical behaviours, as well as examining the 12 individual neurobehavioral summary scores. Hypertonicity and hypotonicity scores were inflated with values of zero and heavily skewed with low frequencies in the 2–5 range. Thus, hypertonicity and hypotonicity were included as binary predictors comparing scores >0 to scores of 0, while the other 10 domains were assessed as continuous predictors of age acceleration.

### Age metrics

PNA for infants in the NOVI study is defined as the time from birth to the time of buccal collection at NICU discharge. PMA is defined as the time from conception to the time of buccal collection. It was calculated by adding PNA to the estimated gestational age that was determined using established methods [15]. For example, an infant who was born at 27 weeks gestational age and had buccal swabs collected at NICU discharge 8 weeks later would have a PNA of 8 weeks and a PMA of 35 weeks.

### Estimates of epigenetic age

Epigenetic age estimates of PMA and PNA were determined using the NEOage epigenetic clocks compatible with the Illumina EPIC array derived from infants in the NOVI cohort [15]. Thus, the algorithms for age prediction were constructed within the same cohort which can cause overfitting, particularly for prediction. To limit this issue, we implemented a leave-one-out strategy, where each participant’s predicted age was based on a training data set that excluded them and their siblings. We also aimed to assess consistency with established clocks and derived epigenetic age estimates using the Horvath skin-blood [21] and PedBE [14] epigenetic clocks, since these clocks did include epithelial and early childhood samples in their training data. For all four clocks, epigenetic age acceleration was calculated and was defined as the residuals when epigenetic age, or predicted age, is regressed on chronological age in an unadjusted linear model.

### Statistical analyses

To assess relationships between age acceleration (dependent variable) and the neonatal morbidity risk score, the individual morbidities, neurobehavioral profiles, and neurobehavioral summary scores, we utilized generalized estimating equations (GEE). GEE models nested infants by family to account for multiple births and adjusted for demographics including infant sex, race, maternal age, education, obesity, and smoking based on our prior work in this cohort and reviews of literature; we also included PMA or PNA as covariates for the PMA and PNA acceleration models, respectively [16,18,22,28,29]. Interaction terms with sex were added to models as a secondary analysis to identify differences in effect by sex.

For our primary analyses, age acceleration was regressed as a continuous dependent variable. However, we also hypothesized that relationships with age acceleration may not be linear, and associations may be more pronounced when focusing on the most and least accelerated ages. Thus, we performed secondary analyses where age acceleration was treated as a binary variable, comparing infants in the top and bottom quintiles of age acceleration to the remaining cohort. We also performed a sensitivity analysis, examining models with the addition of gestational age as a potential confounder due to the uncertain temporality of age acceleration, neurobehavioral characteristics, and neonatal morbidities experienced during NICU stay. A heatmap of the z-scores comparing the NEOage PMA and PNA clocks, Horvath skin-blood clock, and PedBE clock was produced to compare model results across established clocks. All statistical analyses were conducted in R version 4.1.2. Generalized estimating equations were carried out using the *gee* package, and forest plots were produced using the *ggplot* package.

## Results

### Study population

The characteristics of the NOVI cohort have been described in detail elsewhere [22,28]. Of the 542 infants with estimates of epigenetic age, 519 (95.8%) reported data on the covariates of interest and were included in our analyses. The mean (SD) gestational age among the infants was 27.0 weeks (1.9), mean postnatal age was 12.2 weeks (4.5), and mean postmenstrual age was 39.2 weeks (3.4). Postmenstrual age acceleration ranged from −4.79 to 4.86 weeks, and postnatal age acceleration ranged from −9.57 to 3.82 weeks. A total of 189 (36.4%) infants experienced 1 of the 4 morbidities, 98 (18.9%) experienced 2, and 28 (5.4%) experienced 3 or 4. Across the individual morbidities, 67 infants (12.9%) had a brain injury, 34 (6.6%) had severe retinopathy of prematurity, 101 (19.5%) had a serious infection, 126 (24.3%) had mild BPD, and 143 (27.6%) had moderate-severe BPD. The NNNS Profile 6 had the fewest number of infants compared to other profiles with only 35 (6.6%), while Profile 5 consisted of 115 (22.2%) infants. Additional sample characteristics are summarized in Table 1.

**Table 1.** Demographic characteristics of NOVI infants included in analyses; PNA and PMA are in reference to the age at which buccal samples were collected for epigenomic screening.

	Overall (*n* = 519)
**Gestational age at birth** (weeks)	
Mean (SD)	27.0 (1.91)
Median [Min, Max]	27.1 [22.0, 29.9]
**Postnatal age (PNA)** (weeks)	
Mean (SD)	12.2 (4.49)
Median [Min, Max]	11.4 [2.7, 25.3]
**Postmenstrual age (PMA)** (weeks)	
Mean (SD)	39.2 (3.39)
Median [Min, Max]	38.6 [32.1, 51.4]
**Infant Sex**	
Female	228 (43.9%)
Male	291 (56.1%)
**Infant Race/Ethnicity**	
White/Non-Hispanic	225 (43.4%)
White/Hispanic	45 (8.7%)
Other/Non-Hispanic	179 (34.5%)
Other/Hispanic	70 (13.5%)
**Maternal Education**	
Below HS/GED	73 (14.1%)
HS/GED or Higher	446 (85.9%)
**Maternal Smoking Status**	
Yes	72 (13.9%)
No	447 (86.1%)
**Maternal Obesity**	
Yes	183 (35.3%)
No	336 (64.7%)
**Maternal Age** (years)	
Mean (SD)	29.1 (6.37)
Median [Min, Max]	28.5 [17.3, 50.3]

### Continuous PMA acceleration

Infants with a neonatal morbidity risk score of 1 ($\beta_{1}$ = 0.33, p-value = 0.007), 2 ($\beta_{2}$ = 0.45, p-value = 0.008), or 3+ ($\beta_{3}$ = 0.64, p-value = 0.009) were observed to have higher PMA acceleration compared to infants with no morbidities. In addition, infants with mild BPD were observed to have higher PMA acceleration ($\beta_{1}$ = 0.58, p-value = 5.4e-6) compared to infants without BPD, as were infants with moderate-severe BPD ($\beta_{2}$ = 0.78, p-value = 1.1e-6). There were no associations with the other neonatal morbidities, neurobehavioral profiles, or neurobehavioral summary scores (Table 2).

**Table 2.** Regression coefficients and p-values for models examining associations between neonatal morbidity risk score, individual comorbidities, neurobehavioral profiles, and NNNS summary scores with PMA and PNA age acceleration.

	Postmenstrual Age Acceleration		Postnatal Age Acceleration	
	$\beta_{x}$	p-value	$\beta_{x}$	p-value
**Neonatal morbidity risk score**				
1 vs 0	**0.33**	**0.007**	**0.32**	**0.03**
2 vs 0	**0.45**	**0.008**	**0.50**	**0.02**
3+ vs 0	**0.64**	**0.009**	0.12	0.69
**Bronchopulmonary dysplasia**				
Mild vs absent	**0.58**	**5.4e-6**	0.28	0.11
Moderate-severe vs absent	**0.78**	**1.1e-6**	**0.66**	**0.002**
**Serious neonatal infection**				
Present vs absent	0.03	0.82	0.24	0.15
**Severe retinopathy of prematurity**				
Present vs absent	0.27	0.19	0.10	0.72
**Severe brain injury**				
Present vs absent	−0.06	0.73	−0.28	0.21
**NNNS profiles**				
Profile 5 vs Profiles 1–4	0.002	0.99	0.14	0.39
Profile 6 vs Profiles 1–4	0.07	0.76	0.34	0.21
**NNNS summary scores**				
Hypertonicity	0.12	0.30	**0.41**	**0.001**
Hypotonicity	0.04	0.76	0.06	0.72
Nonoptimal reflexes	0.02	0.43	0.006	0.86
Asymmetric reflexes	−0.05	0.20	−0.09	0.08
Quality of movement	0.02	0.77	−0.03	0.73
Attention	−0.07	0.08	0.01	0.76
Handling	−0.01	0.96	−0.20	0.40
Arousal	0.07	0.38	0.11	0.24
Excitability	0.03	0.25	0.05	0.12
Lethargy	0.004	0.87	−0.03	0.30
Regulation	−0.05	0.49	−0.09	0.37
Stress abstinence	0.14	0.86	0.18	0.85

Models were adjusted for sex, site, maternal education, maternal obesity status, maternal smoking status, maternal age, site, PMA or PNA for PMA and PNA AA models, respectively, and clustered by family to account for siblings; Each exposure was assessed in a separate model; $\beta_{x}$ denotes the regression coefficients for the main effects.

### Continuous PNA acceleration

**Figure 1.**
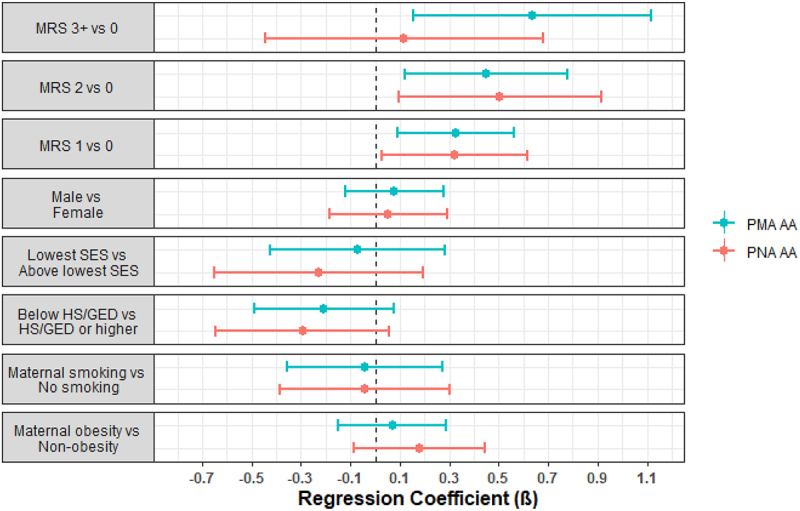
Forest plots of regression parameter estimates for PMA and PNA age acceleration for morbidity risk score (MRS). Models were also adjusted for site, race/ethnicity, maternal age, PMA or PNA for PMA and PNA AA models, respectively, and clustered by family to account for siblings.

**Figure 2.**
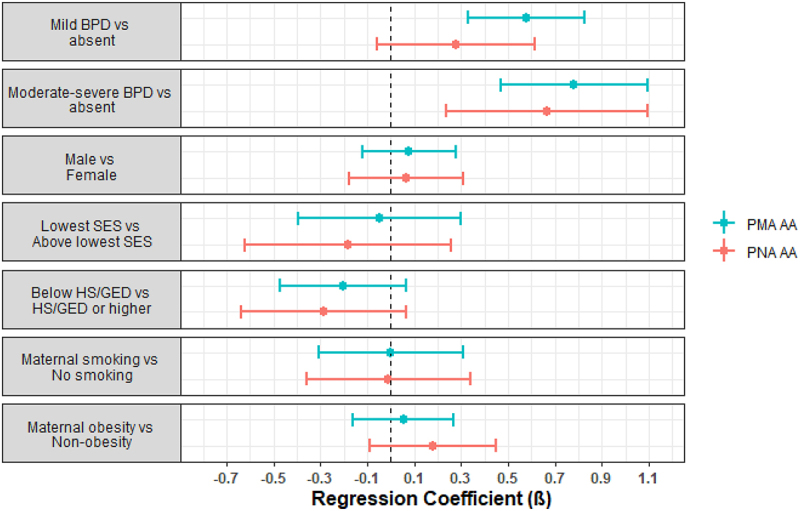
Forest plots of regression parameter estimates for PMA and PNA age acceleration for bronchopulmonary dysplasia (BPD). Models were also adjusted for site, race/ethnicity, maternal age, PMA or PNA for PMA and PNA AA models, respectively, and clustered by family to account for siblings.

We observed similar findings from models regressing PNA acceleration on these morbidities and neurobehavioral characteristics (Table 2, Figures 1–2), though morbidity risk score of 3+ vs 0 ($\beta_{3}$ = 0.12, p-value = 0.69) and mild BPD vs no BPD ($\beta_{1}$ = 0.28, p-value = 0.11) were not statistically significant. Infants with hypertonicity were observed to have higher PNA acceleration compared to infants without hypertonicity ($\beta_{1}$ = 0.41, p-value = 0.001).

### Interaction by sex

We then tested for interaction of the primary covariates with sex, to explore whether there were sex-specific associations with age acceleration. With the addition of an interaction term for sex, infants with moderate-severe BPD were still observed to have both higher PMA acceleration ($\beta_{2}$ = 0.91, p-value = 8.3e-7) and higher PNA acceleration ($\beta_{2}$ = 0.95, p-value = 1.1e-4) compared to infants without BPD, although males with moderate-severe BPD exhibited a decrease in PNA acceleration ($\gamma_{2}$= −0.57, p-value = 0.046) compared to females with moderate-severe BPD. There were no statistically significant interactions with sex in models examining the other neonatal morbidities or neurobehavioral profiles (Appendix, Table 1A).

### Extremes for PMA and PNA acceleration

We also hypothesized that relationships between age acceleration may not be linear and instead may influence whether a neonate is at one of the extreme ends of the age acceleration distribution. Thus, we tested whether these morbidities and behavioural characteristics were related to the odds of being in the top or bottom quintiles for PMA and PNA acceleration. Infants with a neonatal morbidity risk score of 1, 2, or 3+ tended to be more likely to be in the top quintile for both PMA and PNA acceleration, while only those with a morbidity score of 2 yielded statistically significant ORs (PMA OR = 2.33, p-value = 0.02; PNA OR = 2.89, p-value = 0.009) and these infants were less likely to have a PNA acceleration in the bottom quintile (OR = 0.27, p-value = 0.006). Infants with moderate-severe BPD were consistently more likely to be in top quintiles and less likely to be in the bottom quintiles of both PMA and PNA acceleration (Table 3). Among the neurobehavioral summary scores, hypertonicity was associated with lower odds (OR = 0.35, p-value = 8.7e-4) of having a PNA acceleration in the bottom quintile, whereas a higher asymmetric reflexes score was associated with higher odds (OR = 1.28, p-value = 0.008) of being in the bottom quintile for PNA acceleration. An increasing asymmetric reflexes score was also associated with lower odds (OR = 0.80, p-value = 0.04) of having a PMA acceleration in the top quintile. There were no statistically significant associations for the other covariates (Table 3). Overall, these findings generally aligned with what we observed for associations with continuous measures of age acceleration, particularly for the morbidity risk score, BPD, and hypertonicity, while this approach did reveal potential associations between age acceleration and asymmetric reflexes.

**Table 3.** Odds ratios and p-values for models examining associations between neonatal morbidity risk score, individual comorbidities, neurobehavioral profiles, and NNNS summary scores with the top and bottom quintiles of PMA and PNA age acceleration.

	Post-Menstrual Age Age Acceleration				Post-Natal Age Age Acceleration			
	Top Quintile		Bottom Quintile		Top Quintile		Bottom Quintile	
	OR	p-value	OR	p-value	OR	p-value	OR	p-value
**Neonatal morbidity risk score**								
1 vs 0	1.75	0.05	0.85	0.59	1.75	0.07	0.64	0.14
2 vs 0	**2.33**	**0.02**	0.45	0.05	**2.89**	**0.009**	**0.27**	**0.006**
3+ vs 0	1.77	0.32	0.30	0.07	1.58	0.47	0.75	0.61
**Bronchopulmonary dysplasia**								
Mild vs absent	**2.69**	**0.001**	**0.41**	**0.006**	1.93	0.06	0.60	0.13
Moderate-severe vs absent	**3.23**	**8.2e-4**	**0.35**	**0.005**	**3.06**	**0.005**	**0.47**	**0.047**
**Serious neonatal infection**								
Present vs absent	1.08	0.80	0.95	0.86	1.53	0.15	0.54	0.06
**Severe retinopathy of prematurity**								
Present vs absent	1.09	0.86	0.73	0.53	0.71	0.51	1.11	0.83
**Severe brain injury**								
Present vs absent	0.85	0.63	0.87	0.70	0.99	0.98	1.16	0.66
**NNNS profiles**								
Profile 5 vs Profiles 1–4	0.81	0.51	1.47	0.21	1.28	0.43	1.11	0.73
Profile 6 vs Profiles 1–4	1.13	0.81	0.96	0.93	1.16	0.76	0.43	0.14
**NNNS summary scores**								
Hypertonicity	1.08	0.78	0.79	0.38	1.26	0.39	**0.35**	**8.7e-4**
Hypotonicity	1.12	0.71	0.96	0.89	1.30	0.34	0.85	0.61
Nonoptimal reflexes	0.97	0.63	1.04	0.50	0.98	0.71	1.00	0.97
Asymmetric reflexes	**0.80**	**0.04**	1.01	0.92	0.91	0.32	**1.28**	**0.008**
Quality of movement	0.92	0.68	1.02	0.93	0.85	0.34	0.85	0.32
Attention	0.95	0.54	1.04	0.59	1.05	0.63	1.02	0.86
Handling	1.67	0.25	1.52	0.35	0.73	0.51	1.18	0.70
Arousal	1.25	0.24	0.90	0.55	1.14	0.47	0.78	0.22
Excitability	1.12	0.06	0.95	0.35	1.02	0.71	0.91	0.15
Lethargy	0.95	0.37	1.01	0.86	0.94	0.29	1.03	0.58
Regulation	0.87	0.40	0.93	0.67	0.99	0.97	1.05	0.77
Stress abstinence	2.11	0.69	0.27	0.48	0.15	0.31	0.24	0.46

Models were adjusted for sex, site, maternal education, maternal obesity status, maternal smoking status, maternal age, site, PMA or PNA for PMA and PNA AA models, respectively, and clustered by family to account for siblings; Each exposure was assessed in a separate model; OR = Odds Ratio.

### Adjustment for gestational age at birth

We also examined whether the associations between morbidities and behavioural characteristics were potentially driven by upstream differences in gestational age at birth. Infants with shorter gestational ages experience more severe illnesses and are more prone to less optimal neurobehavioral characteristics. Thus, we added gestational age at birth to all models as a sensitivity analysis, and assessed changes in parameter estimates. In all analyses, gestational age was a significant predictor of PNA and PMA acceleration (p-values <0.05), with direction varying across models, and parameter estimates for associations with morbidities were attenuated towards the null, primarily for the PMA acceleration results. Only moderate-severe BPD remained associated with PMA-acceleration, while the morbidity risk score, moderate-severe BPD, and hypertonicity were still associated with PNA acceleration (Table 2A).

### Comparison to other epigenetic clocks

**Figure 3.**
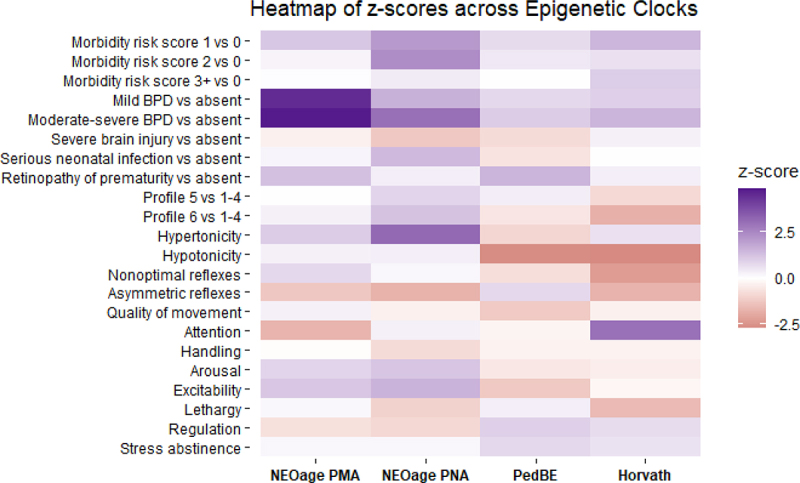
Heatmap of z-scores across epigenetic clocks. Models were adjusted for sex, site, maternal education, maternal obesity status, maternal smoking status, maternal age, site, and clustered by family to account for siblings. NEOage PMA models were also adjusted for PMA, and the three others adjusted for PNA.

Finally, we compare whether we would observe similar associations with PNA acceleration, if we derived age acceleration using the Horvath skin-blood and PedBE clocks. We ran the same models with the primary covariates, controlling for confounders, and compared the parameter estimates to those obtained via models with the NEOage clocks. Figure 3 displays a heatmap of the z-scores for the different models across the four clocks. Overall, the individual morbidities and the neurobehavioral characteristics yielded different directions and magnitudes of association depending on whether the NEOage clocks, Horvath Skin-Blood clock, or PedBE clock were used to estimate age acceleration, but these were also generally null associations. Across all clocks, higher morbidity risk score, and increased BPD severity was associated with increased age acceleration, but these effects were more pronounced when the NEOage clocks were used.

## Discussion

Our analysis indicated that among a cohort of very preterm infants, having any neonatal morbidities and especially mild or moderate-severe BPD was associated with higher PMA and PNA acceleration, with some evidence of these relationships differing by sex for PNA acceleration. Hypertonicity was also associated with PNA acceleration. When we examined differences in associations at the extremes of PMA and PNA acceleration, the morbidity risk score, moderate/severe BPD, and hypertonicity remained associated with age acceleration, while infants with asymmetric reflexes were more likely to have decreased age acceleration. The overall trends in association with the models examining neonatal morbidity risk score and BPD indicate that BPD may be driving the observed association with the morbidity risk score, as the other morbidities did not have a clear relationship with PMA or PNA acceleration when examined independently. This pronounced impact of BPD is similar to what we have previously observed for associations between neonatal multi-morbidities and DNA methylation levels [28].

Lower gestational age at birth is associated with an increased risk of developing adverse health outcomes, as well as differences in neurobehavior. However, the connection to age acceleration is unclear. To explore how adjustment for gestational age at birth might impact our findings, we performed a secondary analysis with the addition of gestational age as a potential confounder. Overall, adjustment for gestational age resulted in attenuation of associations with PMA acceleration, but had no impact on the associations with PNA acceleration. Differences in parameter estimates after adjustment for gestational age were apparent in infants with any morbidities, as the association with PMA acceleration was no longer statistically significant. Additionally, adjustment for gestational age altered the strength of association with BPD and age acceleration in some of the models. These notable changes indicate that timing of birth may set a trajectory for epigenetic ageing, so adjustment for gestational age in our models may greatly alter the conditional effect of other predictor variables on age acceleration.

A study by Knight et al. [30] found that higher gestational age acceleration, estimated from cord blood, was associated with decreased risk of BPD among extremely preterm infants, which is opposite in direction to what we observed. However, Knight et al. focused on gestational age acceleration at birth, whereas our focus was on PMA and PNA acceleration measured at NICU discharge. Thus, their observation that decreased gestational age acceleration was associated with increased risk of BPD may be an indicator of biological immaturity at birth. However, with PNA and PMA acceleration in our study, it is unclear whether the age acceleration preceded or is a consequence of the experienced morbidities. It is possible that our PNA and PMA acceleration metrics are capturing the stress experienced during the NICU stay, including the NICU environment and the medical procedures that are utilized to treat these morbidities. This would align with other research that demonstrates a relationship between early-life stress, adversity [31,32], and major medical procedures [33] with age acceleration. Additionally, in agreement with our findings, in Knight et al. [30] males with BPD had lower age acceleration compared to females with BPD. Another small study (*N* = 35) showed that infants with BPD had accelerated ageing as estimated from the PedBE clock but with a p-value <0.12; while not statistically significant, the direction of effect aligns with our findings, and the small sample size was likely underpowered to detect small effect sizes. Gomaa et al. [34] also found that age acceleration was significantly associated with other neonatal morbidities, including severe retinopathy of prematurity and patent ductus arteriosus, and with poorer performance on prospectively collected Bayley cognitive scores collected at 18 months of age [34]. Thus, all three studies indicate that there is a relationship between BPD, and possibly other neonatal morbidities, and neonatal age acceleration, which may differ by timing of tissue collection, tissue type, and sex of the newborn or infant. However, the findings across these studies indicate that the utility of age acceleration as a biomarker of developmental maturity is not yet clear in infants born preterm, highlighting the need for further research to better establish the relationships and temporality of gestational age at birth, neonatal morbidities, and age acceleration.

In addition to the Knight et al. gestational age clock and the PedBE paediatric epigenetic clock, Bohlin et al. [35] and Haftorn et al. [36,37] have produced epigenetic gestational age clocks to help investigate how perinatal age acceleration associates with prenatal exposures and children’s health and development. The recently developed clock by Haftorn et al. [36] also showed improved performance in preterm newborns and thus may allow for new opportunities to study age acceleration in this high-risk population. To date, these clocks have not been studied in relation to the neonatal morbidities and neurobehavioral responses that we focused on, and thus we could not make direct comparisons to our results. However, all of these perinatal and paediatric epigenetic clocks highlight a growing area of study that aims to better understand how early life age acceleration relates to prenatal exposures and children’s health and development.

We did not observe statistically significant associations of the two atypical neurobehavioral profiles with PMA or PNA acceleration when compared to the rest of the NOVI infants. This aligns with a prior study of ours, where we found no significant association with age acceleration when comparing profile 6 to the remaining neurobehavioral profiles using the Horvath clock [28]. However, in the current study, we did identify statistically significant associations between age acceleration and hypertonicity in both primary and secondary analyses, and with asymmetric reflexes in secondary analyses, but these associations were in opposite directions. Other research has linked physical and cognitive functions [38], which both relate to neurobehavior, with differences in age acceleration.

Analyses examining the PedBE and Horvath-Skin blood epigenetic clocks revealed considerable differences in z-scores across the different models. These results were not surprising, as the PedBE clock was derived using buccal epithelial cells from individuals 0–20 years old [14] and the Horvath skin-blood clock was derived from individuals of a wider age range, and DNA was extracted from a wider array of samples [21]. Correlations between predicted and actual PMA and PNA were also considerably lower using these two epigenetic clocks when applied to the NOVI cohort [15]. The reduced accuracy of the Horvath and PedBE clocks is likely due to two factors. First, the age ranges of our cohort are at (or even outside of) the boundaries of the training data used to derive these clocks, which is why our group derived the NEOage clocks in the first place. Second, the NEOage clocks were derived in the NOVI cohort, and thus the algorithm undoubtedly overfits to our dataset. We aimed to reduce the impact of this overfitting by using leave-one-out analysis described in detail in our prior publication [15]. Briefly, each infant’s estimate of epigenetic age was calculated based on an algorithm that excluded their own and their sibling’s epigenetic data from the training algorithm. While this reduces some of the overfitting issue, an improved version of the NEOage clocks would integrate additional epigenetic data from additional populations of infants born preterm across a range of postnatal ages during this critical developmental period.

Although our analyses revealed some associations between age acceleration, neonatal morbidities, and neurobehavioral domains, given the rapidly changing and intricate biological mechanisms occurring in infants, our findings may not be as readily applicable if extrapolated to infants not born very preterm. The strongest and most consistent associations were observed with moderate to severe BPD, which primarily impacts very and extremely preterm newborns. Given that the relationship between neonatal age acceleration, early morbidities, and neurobehavior has only been studied in a few other small sample cohorts, we aimed to avoid Type-II errors and did not apply a strict multiple testing correction [39]. However, we primarily emphasize our findings of accelerated epigenetic age with BPD as this finding would remain significant even with Bonferroni-correction and this result aligns with the findings from other similar studies. Another important limitation of our findings is that DNA samples were collected shortly before infants were discharged from the NICU and to be included in the study infants had to survive to NICU discharge, introducing potential survivorship bias into our results. The observed association with BPD, for example, may indicate that infants exhibiting accelerated growth patterns were infants more likely to survive their lung complications, leading to the associations we observed.

Our findings indicate that neonatal morbidities, particularly BPD, and some neurobehavioral characteristics may be associated with age acceleration even among early neonates. Further research may consider collecting DNAm at birth and NICU discharge to assess for differences among infants surviving and those who succumb to their adverse health outcomes, in addition to allowing for better establishment of the temporality of age acceleration and neonatal morbidities and neurobehavior. Doing so may improve our understanding of the use of age acceleration as a biomarker of developmental maturity for this vulnerable population and potentially identify at-risk neonates for preventative interventions to improve health outcomes.

## Data Availability

The genome-wide epigenetic data that were used to derive epigenetic age are accessible through NCBI Gene Expression Omnibus (GEO) via accession series GSE128821.

## Appendix

**Table A1.** Regression coefficients and p-values for models examining associations between neonatal morbidity risk score, individual comorbidities, neurobehavioral profiles, and NNNS summary scores with PMA and PNA age acceleration, including interaction by sex. Models were adjusted for sex, site, maternal education, maternal obesity status, maternal smoking status, maternal age, site, PMA or PNA for PMA and PNA AA models, respectively, and clustered by family to account for siblings; Each exposure was assessed in a separate model; βx denotes the regression coefficients for the main effects; γx denotes sex interaction term, where female is the reference category.

	Post-Menstrual Age Age Acceleration				Post-Natal Age Age Acceleration			
	$\beta_{x}$	p-value	$\gamma_{x}$	p-value	$\beta_{x}$	p-value	$\gamma_{x}$	p-value
**Neonatal morbidity risk score**								
1 vs 0	0.29	0.09	0.06	0.77	**0.44**	**0.04**	−0.22	0.41
2 vs 0	0.47	0.05	−0.04	0.90	**0.76**	**9.7e-3**	−0.47	0.21
3+ vs 0	0.63	0.04	0.02	0.97	0.24	0.52	−0.22	0.65
**Bronchopulmonary dysplasia**								
Mild vs absent	**0.50**	**0.02**	0.12	0.65	0.37	0.20	−0.18	0.60
Moderate-severe vs absent	**0.91**	**8.3e-7**	−0.28	0.22	**0.95**	**1.1e-4**	**−0.57**	**0.046**
**Serious neonatal infection**								
Present vs absent	−0.002	0.99	0.07	0.79	0.35	0.13	−0.22	0.45
**Severe retinopathy of prematurity**								
Present vs absent	0.16	0.57	0.24	0.56	0.008	0.98	0.19	0.72
**Severe brain injury**								
Present vs absent	−0.13	0.61	0.14	0.67	−0.41	0.28	0.25	0.58
**NNNS profiles**								
Profile 5 vs Profiles 1–4	−0.06	0.76	0.11	0.64	0.17	0.49	−0.04	0.88
Profile 6 vs Profiles 1–4	0.10	0.77	−0.05	0.91	0.22	0.60	0.19	0.68
**NNNS summary scores**								
Hypertonicity	0.23	0.17	−0.20	0.34	**0.49**	**9.6e-3**	−0.14	0.55
Hypotonicity	−0.25	0.23	0.48	0.08	−0.25	0.42	0.51	0.16
Nonoptimal reflexes	−0.0006	0.99	0.04	0.37	−0.01	0.78	0.04	0.52
Asymmetric reflexes	0.007	0.90	−0.12	0.14	−0.02	0.80	−0.14	0.15
Quality of movement	0.08	0.47	−0.10	0.47	0.09	0.48	−0.20	0.22
Attention	−0.07	0.22	−0.007	0.92	0.008	0.89	0.01	0.88
Handling	0.30	0.31	−0.54	0.16	0.41	0.27	**−1.08**	**0.02**
Arousal	0.19	0.08	−0.23	0.15	**0.27**	**0.04**	−0.30	0.09
Excitability	0.07	0.048	−0.07	0.15	0.05	0.25	−0.006	0.92
Lethargy	−0.007	0.87	0.02	0.68	−0.04	0.33	0.02	0.67
Regulation	−0.05	0.64	−0.008	0.95	0.03	0.84	−0.22	0.15
Stress abstinence	0.48	0.67	−0.55	0.68	0.81	0.57	−1.03	0.52

Models were adjusted for sex, site, maternal education, maternal obesity status, maternal smoking status, maternal age, site, PMA or PNA for PMA and PNA AA models, respectively, and clustered by family to account for siblings; Each exposure was assessed in a separate model; βx denotes the regression coefficients for the main effects; γx denotes sex interaction term, where female is the reference category.

**Table A2.** Regression coefficients and p-values for models examining associations between neonatal morbidity risk score, individual comorbidities, neurobehavioral profiles, and NNNS summary scores with PMA and PNA age acceleration, adjusting for gestational age.

	Post-Menstrual Age Age Acceleration		Post-Natal Age Age Acceleration	
	$\beta_{x}$	p-value	$\beta_{x}$	p-value
**Neonatal morbidity risk score**				
1 vs 0	0.13	0.25	**0.30**	**0.046**
2 vs 0	0.04	0.81	**0.50**	**0.02**
3+ vs 0	0.01	0.95	0.19	0.51
**Bronchopulmonary dysplasia**				
Mild vs absent	0.24	0.05	0.33	0.05
Moderate-severe vs absent	**0.45**	**0.005**	**0.63**	**0.004**
**Serious neonatal infection**				
Present vs absent	−0.04	0.76	0.21	0.21
**Severe retinopathy of prematurity**				
Present vs absent	−0.14	0.46	0.21	0.45
**Severe brain injury**				
Present vs absent	−0.19	0.23	−0.27	0.22
**NNNS profiles**				
Profile 5 vs Profiles 1–4	−0.05	0.73	0.16	0.34
Profile 6 vs Profiles 1–4	0.006	0.98	0.33	0.22
**NNNS summary scores**				
Hypertonicity	0.25	0.15	**0.34**	**0.008**
Hypotonicity	−0.02	0.89	0.09	0.60
Nonoptimal reflexes	−0.008	0.77	0.02	0.66
Asymmetric reflexes	−0.07	0.07	−0.09	0.08
Quality of movement	0.05	0.46	−0.05	0.58
Attention	−0.002	0.96	−0.02	0.68
Handling	−0.02	0.91	−0.17	0.48
Arousal	0.10	0.17	0.08	0.37
Excitability	0.03	0.21	0.04	0.17
Lethargy	−0.03	0.26	−0.01	0.61
Regulation	−0.04	0.60	−0.09	0.40
Stress abstinence	−0.19	0.81	0.27	0.78

Models were adjusted for sex, site, maternal education, maternal obesity status, maternal smoking status, maternal age, site, gestational age, PMA or PNA for PMA and PNA AA models, respectively, and clustered by family to account for siblings; Each exposure was assessed in a separate model; $\beta_{x}$ denotes the regression coefficients for the main effects.
